# Early life influences on the development of food addiction in college attending young adults

**DOI:** 10.1007/s40519-023-01546-3

**Published:** 2023-02-20

**Authors:** Rachel A. Wattick, Melissa D. Olfert, Elizabeth Claydon, Rebecca L. Hagedorn-Hatfield, Makenzie L. Barr, Cassie Brode

**Affiliations:** 1 Division of Animal and Nutritional Sciences, Davis College of Agriculture, Natural Resources, and Design, West Virginia University, G25 4100 Agricultural Sciences Building, 1194 Evansdale Dr., P.O. Box 6108, Morgantown, WV 26505-6108 USA; 2 Department of Social and Behavioral Sciences, School of Public Health, West Virginia University, 64 Medical Center Dr., Morgantown, WV 26505-9190 USA; 3 Department of Nutrition, Health and Human Performance, School of Education Health and Human Sciences, Meredith College, 3800 Hillsborough Street, Raleigh, NC 27607-5298 USA; 4 Department of Dietetics and Human Nutrition, University of Kentucky, 212 Funkhouser Building, Lexington, KY 40514 USA; 5 Department of Behavioral Medicine and Psychiatry, West Virginia University School of Medicine, Morgantown, WV USA

**Keywords:** Food addiction, College student, Young adult, Mixed-methods, ACEs

## Abstract

**Purpose:**

There is little investigation into the causes of food addiction. The aim of this study was to determine the impact of early life influences on the development of food addiction in college-attending young adults aged 18–29.

**Methods:**

This study utilized a sequential explanatory mixed-methods research design. College-attending young adults were invited to complete an online survey measuring Adverse Childhood Experiences (ACEs), food addiction, depression, anxiety, stress, and demographic information. Correlations between food addiction and the other variables were analyzed and significant variables were placed into a nominal logistic regression model to predict the development of food addiction. Participants who met the criteria for food addiction were invited to participate in interviews to examine their childhood eating environment and when their symptoms emerged. Interviews were transcribed and thematically analyzed. Quantitative analysis was conducted using JMP Pro Version 16.0 and qualitative analysis was conducted using NVIVO Software Version 12.0.

**Results:**

Survey respondents (*n* = 1645) had an overall 21.9% prevalence of food addiction. Significant correlations were observed between food addiction and ACEs, depression, anxiety, stress, and sex (*p* < .01 for all). Depression was the only significant predictor of the development of food addiction (OR = 3.33 95% CI 2.19, 5.05). The most common eating environment described by interview participants (*n* = 36) was an emphasis on diet culture, ideal body image, and restrictive environments. Symptoms frequently emerged after transitioning into college and having the ability to make their own food choices.

**Conclusion:**

These results show the impact of early life eating environments and young adulthood mental health on the development of food addiction. These findings contribute to the understanding of underlying causes of food addiction.

*Level of evidence:* Level V, Opinions of authorities, based on descriptive studies, narrative reviews, clinical experience, or reports of expert committees.

## Introduction

Within the United States, approximately 42.4% of individuals are overweight or obese [1]. Despite decades of research on treating obesity, little progress has been made on reducing its prevalence [2]. Obesity is a health problem of concern as it increases an individual’s risk of developing type 2 diabetes, coronary heart disease, end-stage renal disease, and facing psychosocial complications such as weight-related stigma [1, 3]. The focal cause of obesity has been an excess intake of calories through overeating, but more recently the focus has been on biological and environmental factors, or psychosocial factors [2]. Today’s food environment is characterized by the widespread prevalence of highly-palatable foods [4], such as foods with added sugars or highly processed or refined foods, and an increase in the consumption of foods for pleasure rather than sustenance [5]. These societal trends co-occurring with the rise in obesity has led to the examination of the potential addictive properties of food, or the study of food addiction.

Food addiction can be defined as “a chronic and relapsing condition caused by the interaction of many complex variables that increase cravings for certain specific foods in order to achieve a state of high please, energy, or excitement, or to relieve negative emotional or physical states” [6]. Food addiction has shown multiple neurological similarities to drug addiction. Individuals with food addiction display high activation of reward systems when anticipating intake of highly palatable foods. Illicit drugs and food have been shown to cause the same feelings of gratification through activation of the dopamine neurons in the ventral tegmental area followed by release of dopamine in the nucleus accumbens [7]. Emotionally, there are similarities between populations with substance use disorder (SUD) and food addiction, including a lack of emotional clarity, poor impulse control, higher emotional dysregulation, and higher non-acceptance of emotional responses [8].

Within examination of psychosocial causes of obesity, there is a strong body of evidence on adverse childhood experiences (ACEs), which can take the form of emotional, physical, or sexual abuse, neglect, or household dysfunction. [9–13] Additionally, other methods of measuring childhood abuse and trauma have been associated with adulthood obesity [14–16]. From this research, it has been shown that individuals with ACEs can develop mal-adaptive coping strategies to stress and other negative emotions, including overeating [12, 17]. ACEs can disrupt an individual’s chronic stress response by altering the function of the hypothalamic–pituitary–adrenal (HPA) axis, leaving these individuals more vulnerable to high stress and subsequently higher risk for negative coping mechanisms [9]. ACEs have also been shown to lead to adulthood SUD [18, 19]. There are similarities between the populations with SUD and obesity, including reduced sensitivity to reward [20, 21], which can lead to addictive behavior [13]. The growth in food addiction research rapidly increased in 2009 after Gearhardt et al. developed the Yale Food Addiction Scale (YFAS) to diagnose food addiction [22]. It has been investigated as a possible mediator between childhood trauma and adulthood obesity [23]. However, there is still little known about the causes of food addiction. Overall, research on childhood trauma and food addiction has found interesting outcomes in the clinical population, such as bariatric surgery candidates [10, 24] and individuals with eating disorders [25], and in the population of females over age 35 [3, 20]. While this is important, more non-clinical investigations are needed in at-risk populations. Additionally, there is evidence of ACEs leading to the development of eating disorders [26, 27]. Although food addiction is similar to other eating disorders, it is still not entirely the same construct, [7] and therefore needs separate investigation. Additionally, there are other influences on eating behavior and obesity, such as parental feeding style [28] and general parenting style [29]. For example, in order to help their children have a healthy relationship with food, parents are supposed to trust children to determine how much food they should eat and decide to eat from what parents provide [28]. Conversely, too much parental control over child food intake can lead to unhealthy eating patterns and higher weight [30]. Therefore, investigating other early life influences on eating behavior is integral to understanding how food addiction can occur.

College-attending young adults are at a formative period of their lives during which lifelong lifestyle behaviors are established [31–33]. This period can be characterized by high stress, weight gain, and the development of behavioral disorders. Young adults are heavily influenced by the food environment [5] and consistently show unhealthy eating habits [34]. There is a risk of developing food addiction as a response to stress or to cope with negative emotions. Although there have been several investigations into food addiction among college students [5, 34–36], there is little investigation into possible contributing factors. The aim of this study is to determine the impact of ACEs and other early life experiences on the development of food addiction in college-attending young adults. Because it was anticipated that the quantitative data would not entirely capture early life influences on food addiction, qualitative data was also collected to investigate other potential influences.

## Methods

### Study design

This mixed-methods study used a sequential explanatory analysis approach in which quantitative data is collected first, analyzed, and then used to collect qualitative data to contribute to explanation of findings from the quantitative study [37]. This design was chosen in order to be able to further explain potential influences on food addiction that were not explained by quantitative data. To achieve this, a cross-sectional study was first conducted to examine a sample of young adults aged 18–29 attending a large, Appalachian university in fall 2021. Participants were currently enrolled college students. Following quantitative analysis, participants with food addiction were invited to complete in the qualitative portion of semi-structured open-ended interviews to elucidate other causes or contributing factors to their development of food addiction. All subjects gave their written informed consent for inclusion before they participated in the study. The study was conducted in accordance with the Declaration of Helsinki, and the protocol was approved by the Institutional Review Board at West Virginia University (#2106344268).

### Participants and procedures

A convenience sample of undergraduate and graduate students attending a large, land-grant university in central Appalachia was recruited during the fall 2021 semester. A list of emails of all active and registered students during the fall 2021 semester was obtained and students were emailed an invitation to participate that included a Qualtrics link, an online survey platform (Qualtrics, Provo, IT, USA). Exclusion criteria included not being within the young adult age group (ages 18 to 29). Participants were instructed to read the informed consent and if they agreed to participate, they proceeded to complete the survey. Students who denied participation were thanked for their time and exited from the survey. After completing the survey, students were informed that the primary outcome of the study was food addiction and were asked if they would be willing to be contacted again to participate in an interview if they are diagnosed with food addiction based on their responses. If they agreed, participants provided their email for future contact. Students were incentivized to complete the survey by a chance to win one of three $100 American Express gift cards by entering their contact information following survey completion.

### Ethical considerations

Due to the sensitive nature of the survey and interview questions, participants were provided with a counseling services referral list multiple times throughout the investigation. Additionally, participants could choose to end the interview at any time if they were uncomfortable.

### Quantitative approach

#### Survey design

This 97-item survey was developed by the Lifestyle Intervention Research Lab at West Virginia University using validated tools to investigate a breadth of psychosocial and behavioral characteristics of college-attending young adults. The variables utilized for this study were food addiction using the Yale Food Addiction Scale 2.0 (YFAS 2.0) [38], childhood trauma using the Adverse Childhood Experiences (ACE) Questionnaire [39], depression using the Patient-Health Questionnaire-9 Item (PHQ-9) [40], anxiety using the Generalized Anxiety Disorder-7 Item (GAD-7) [41], and stress using Cohen’s Perceived Stress Scale-10 Item (PSS-10) [42]. Demographic information was also collected. The estimated time to complete the survey was 35 min.

#### Dependent variable

##### Food addiction

The YFAS 2.0 is a 35-item validated tool used to diagnose food addiction. It was designed to reflect the diagnostic criteria for substance use disorder in the DSM-5. The YFAS 2.0 measures symptoms of food addiction, such as “I spent a lot of time eating certain foods throughout the day” or “I avoided work, school, or social activities because I was afraid I would overeat there”. Using the diagnostic scoring method, symptom totals are counted. Importantly, the presence of clinically significant impairment or distress is necessary to be diagnosed with food addiction. Using this method, individuals are designated as having no food addiction (1 or fewer symptoms and/or no clinical significance), mild food addiction (2 or 3 symptoms and clinical significance), moderate food addiction (4 or 5 symptoms and clinical significance), or severe food addiction (6 or more symptoms and clinical significance). For analysis, both the symptom count and diagnostic methods were used for descriptive data, and only the diagnostic method was used for correlation analysis and nominal logistic regression. (Cronbach’s *α* = 0.94).

#### Independent variables

##### Childhood trauma

The ACE questionnaire is a 10-item tool that measures an individual’s experience of childhood trauma, such as emotional neglect, physical or sexual abuse, or household dysfunction, during their first 18 years of life. Participants can answer either “yes” or “no” if they experienced each of the 10 events. Affirmative responses are scored as 1 and summed to indicate total ACE scores. Responses can be analyzed in a continuous manner or by using a cut-off of 4 or more ACEs indicating a high number of ACEs. Both methods were computed for descriptive statistics and for correlation and nominal logistic regression analyses, continuous scores were used. (Cronbach’s *α* = 0.77)**.**

##### Depression

The PHQ-9 is a 9-item tool used to measure an individual’s depression symptoms over the prior 2 weeks. Symptoms include “feeling tired or having little energy”, “little interest or pleasure in doing things”, and “trouble concentrating on things, such as reading the newspaper or watching television” with response options of “Not at all”, “Several days”, “More than half the days”, and “Nearly every day”. Scores can be summed and scored from 0 to 27 with higher scores indicating higher symptoms of depression. (Cronbach’s *α* = 0.90).

##### Anxiety

The GAD-7 is a 7-item tool that asks respondents questions on their experience of anxiety symptoms over the prior 2 weeks. Symptoms include items such as “feeling nervous, anxious, or on edge”, “trouble relaxing”, and “becoming easily annoyed or irritable”. Response options include “Not at all”, “Several days”, “More than half the days”, and “Nearly every day”. Scores can be summed for a total of 0 to 21 with higher scores indicating more symptoms of anxiety. (Cronbach’s *α* = 0.92)**.**

##### Stress

The PSS-10 is a scale that contains 10 items on perceived stress over the past month. Items include questions such as “How often have you been upset because of something that happened unexpectedly?” and “How often have you felt that you could not cope with all the things that you had to do?” with response options of “Never”, “Almost never”, “Sometimes”, “Fairly often”, and “Very often”. Scores are summed for a total of 0 to 40 with higher scores indicating higher stress. (Cronbach’s *α* = 0.86)**.**

Demographic variables included assigned sex at birth, gender, age, race/ethnicity, year in school, and height and weight.

#### Statistical analysis

Descriptive statistics were computed for all demographic variables as well as for food addiction, ACEs, depression, anxiety, and stress. BMI (body mass index, kg/m2) was calculated from participants’ self-reported height and weight and categorized using the World Health Organization (WHO) BMI classification guide [43]. For descriptive statistics, YFAS was scored both in a continuous and ordinal fashion. For example, YFAS symptom counts, and diagnoses were computed according to protocol, and diagnoses of mild, moderate, and severe food addiction were computed. Distributions were checked for skewness and kurtosis and variables were log transformed if necessary. The only variable that violated rules for skewness and kurtosis was BMI (skewness = 1.61, kurtosis = 3.67, transformed skewness = 0.79, kurtosis = 0.88), When reporting means, untransformed means were used to reflect the meaningful values of the BMI scale.

For bivariate analysis, a diagnosis of no food addiction was computed as 0, and diagnoses of mild, moderate, and severe food addiction were grouped together and designated as 1. Simple logistic fit was used to determine significant associations between food addiction and continuous sociodemographic and psychosocial variables and Chi-square was used for significant associations between categorical sociodemographic variables. All significant variables from this analysis were placed into a full logistic regression model to predict food addiction. Data were analyzed using JMP Pro Version 16.0. Significance criterion alpha for all tests was 0.05.

### Qualitative approach

#### Development of interview questions

Interview questions were developed by the Lifestyle Intervention Research Lab at West Virginia University. The questionnaire followed a semi-structured, open-ended question format. Questions were generated based on aspects of the survey that needed further exploration. The entire questionnaire contained 13 questions with 9 probes. The questions analyzed for this study were under the topic of “Early Life Influences” and “Emergence of Symptoms”. Below are the questions used for this analysis.

1. Please describe to me your eating environment growing up, family attitudes towards food, family meal practices, or anything related to that.
2. Around what time in your life did you notice these symptoms start to appear?
  - Probe: What was going on around that time that you feel could have contributed to the emergence of these symptoms?

#### Participants and conducting of interviews

Participants who had a diagnosis of food addiction from their survey responses and who indicated they would be willing to be contacted to participate in another interview were emailed the invitation to participate and schedule an interview via Zoom, a virtual meeting platform. Individuals who agreed to participate signed additional consent before completing the interview. Interviews were audio recorded for transcription purposes.

#### Data analysis

Thematic analysis, which is gleaning major themes and subthemes from qualitative data [44], was used to analyze data. Interviews were transcribed verbatim from the audio recordings. All interviews were reviewed multiple times before coding began. To code data, responses to the questions under the topic of Early Life Influences were reviewed and codes were assigned to responses that had similar qualities. Codes were based on subjective assessment. After coding all transcripts, the codes were reviewed, and additional codes were added if deemed necessary. Similar codes were grouped together to generate themes and subthemes. Review of themes and subthemes then occurred and necessary changes were made. A secondary reviewer reviewed all themes and subthemes, and agreement was reached between both reviewers to generate a final list of themes and subthemes. All themes and subthemes were described and example quotes illustrating the theme were chosen. Qualitative analysis followed a traditional generic thematic analysis approach.[44].

## Results

### Quantitative

Respondents [*n* = 1645] had an overall 21.9% prevalence of food addiction, with 5.7% of that being mild, 4.7% being moderate, and 11.5% being severe. Respondents were primarily female (76.8%), identified as a woman (74.1%), White (84.4%), had a normal-weight BMI (52.9%), and were most commonly in graduate/professional school (27.2%). The mean age of respondents was 22.03 ± 5.15 years. The mean number of YFAS symptoms was 2.01 ± 2.84 and the mean ACE score was 2.05 ± 2.20. There was an Tables 1 and 2 below summarize demographic and psychosocial information.

**Table 1:** Distributions of all demographic variables

Variable	Frequency (*N*) (%)
Sex	
Male	361 (23.2)
Female	1196 (76.8)
Gender Identity	
Man	352 (22.6)
Woman	1153 (74.1)
Nonbinary	45 (2.9)
Other/Self-describe	7 (0.5)
Race	
White/Caucasian	1313 (84.4)
Black/African American	40 (2.6)
Asian or Pacific Islander	62 (4.0)
Hispanic or Latino	34 (2.1)
Multiracial or Biracial	91 (5.9)
Native American or Alaska Native	2 (0.1)
Race/ethnicity not listed here	13 (0.8)
Grade	
Freshman	275 (17.7)
Sophomore	256 (16.4)
Junior	285 (18.3)
Senior	317 (20.4)
Graduate/Professional School	424 (27.2)
BMI Categories	
Underweight [< 18.5 kg/m^2^]	82 (5.3)
Normal range [18.5–24.9 kg/m^2^]	821 (52.9)
Overweight [25.0–29.9 kg/m^2^]	365 (23.5)
Obese [30.0 + kg/m^2^]	284 (18.3)

Variable	Mean ± SD [95% CI]
Age [Years]	22.03 ± 5.15 (21.8, 22.3)
BMI (kg/m^2^)	25.4 ± 6.1 (25.09, 25.67)

Demographic data represented in frequencies and percentages for categorical variables and in means and standard deviations for continuous variables. *BMI* body mass index (kg/m^2^)

**Table 2:** Distributions of all psychosocial variables

Variable	Frequency (*N*) (%)
Food addiction	
None (≤ 1 symptom or no CS)	1285 (78.1)
Mild (2–3 symptoms and CS)	93 (5.7)
Moderate (4–5 symptoms and CS)	78 (4.7)
Severe (6 + symptoms and CS)	189 (11.5)
ACE Levels	
Low (< 4)	1175 (75.8)
High (≥ 4)	376 (24.2)

Variable	Mean ± SD (95% CI)
YFAS symptoms	2.01 ± 2.84 (1.87, 2.15)
ACE score	2.05 ± 2.20 (1.94, 2.16)
PHQ-9 score	9.46 ± 6.57 (9.14, 9.77)
GAD-7 score	8.60 ± 6.07 (8.31, 8.89)
PSS-10 score	20.3 ± 7.07 (19.98, 20.68)

Psychosocial data represented in frequencies and percentages for categorical variables and in means and standard deviations for continuous variables. *SD* standard deviation, *YFAS* Yale Food Addiction Scale, *CS* Clinical Significance, *ACE* Adverse Childhood Experiences, *PHQ-9* Patient Health Questionnaire 9-Item, *GAD-7* Generalized Anxiety Disorder-7 Item, *PSS-10* Perceived Stress Scale-10 Item, *CI* Confidence Interval

Simple logistic fit showed that ACE scores, depression, anxiety, stress, and sex were significantly associated with food addiction status (p < 0.0001 for all), but age, grade, and race were not significant. All significant variables were placed into a full logistic regression model to predict any food addiction from all significant variables from Pearson’s *r* analysis. Depression was the only significant predictor of the development of food addiction, with each point increase on the depression scale increasing the odds of food addiction by 233% (OR = 3.33 95% CI 2.19, 5.05). Being female was trending towards significance. Results are shown in Table 3.

**Table 3:** Logistic regression model predicting food addiction

Variable	Odds ratio	95% Confidence interval	*P*-value
ACE Score	1.05	(0.82, 1.35)	0.6770
Depression	3.33	(2.19, 5.05)	< 0.0001*
Anxiety	0.98	(0.69, 1.38)	0.8974
Stress	1.03	(0.99, 1.07)	0.1758
Sex	1.59	(0.99, 2.55)	0.0572

Selection criteria for the model entry was p < 0.05. Variables from simple analyses were entered into a nominal logistic regression model. Depression remained a significant predictor of the development of food addiction, with being female trending towards significance. ACE score, anxiety, and stress were not significant predictors. * denotes significance

### Qualitative results

Of the 360 participants in the survey who had a diagnosis of food addiction, 241 agreed to be contacted for an interview. Of the 241 emails sent, 40 responded to schedule an interview time. Four participants did not show up for their interviews, resulting in 36 interviews being conducted.

### Interview participant characteristics

Interview participants were mostly diagnosed as having severe food addiction (72.2%), with 11.1% having moderate and 16.7% having mild. The mean ACE score was 2.61 ± 2.16, with 30.6% having a high number of ACEs, and the mean number of YFAS symptoms was 6.75 ± 2.71. Most participants were female (80.6%), White (88.9%) and in graduate/professional school (30.6%). The mean age of participants was 22.1 ± 4.63 years, and the mean BMI was 29.7 ± 8.30 kg/m^2^, with most (36.1%) falling into the obese category. Interview participant characteristics are reported in Table 4.

**Table 4:** Distributions of demographic and psychosocial variables of interview participants

Variable	Frequency (*N*) (%)
Food addiction	
Mild (2–3 symptoms and CS)	6 (16.7)
Moderate (4–5 symptoms and CS)	4 (11.1)
Severe (6 + symptoms and CS)	26 (72.2)
Sex	
Male	7 (19.4)
Female	29 (80.6)
Race	
White/Caucasian	32 (88.9)
Asian or Pacific Islander	2 (5.6)
Hispanic or Latino	1 (2.8)
Native American or Alaska Native	1 (2.8)
Grade	
Freshman	4 (11.1)
Sophomore	10 (27.8)
Junior	3 (8.3)
Senior	8 (22.2)
Graduate/Professional School	11 (30.6)
BMI Category	
Underweight (< 18.5 kg/m^2)^	2 (5.6)
Normal range (18.5–24.9 kg/m^2^)	9 (25.0)
Overweight (25.0–29.9 kg/m^2^)	12 (33.3)
Obese (30.0 + kg/m^2^)	13 (26.1)
ACE Levels	
Low (< 4)	69.4
High (≥ 4)	30.6

Variable	Mean ± SD (95% CI)
YFAS Symptoms	6.75 ± 2.71 (5.83, 7.67)
Age (years)	22.1 ± 4.63 (20.5, 23.76)
ACE Score	2.61 ± 2.16 (1.88, 3.34)
BMI [kg/m^2^]	29.7 ± 8.30 [26.90, 32.51]

Demographic and psychosocial data represented in frequencies and percentages for categorical variables and in means and standard deviations for continuous variables. *SD* standard deviation, *YFAS* Yale Food Addiction Scale, *CS* Clinical Significance, *ACE* Adverse Childhood Experiences, *CI* Confidence Interval, *BMI* Body Mass Index

## Themes: early life influences and emergence of symptoms

The topic of “Early Life Influences” generated 4 themes. Participants were asked about their eating environment growing up, including their family meal practices and attitudes towards food. Participants were asked around what time in their life their symptoms started to occur and what was going on in their life around that time. Responses to these questions under the topic of “Emergence of Symptoms” generated 5 themes and 1 subtheme. All themes are described with example quotes given in Table 5.

**Table 5:** Summary of Themes and Subthemes for Early Life Influences and Emergence of Symptoms

Topic	Theme(s)	Subtheme(s)	Description	Example quote
Early life influences	Pressure to be Healthy (*n* = 29)	Diet Culture in Family Restrictive Environment Body Image Pressures	Participants expressed growing up in environments that had an emphasis on an ideal body weight or physique. They often were restricted on what foods they were allowed to eat, with parents or other role models modeling restrictive behaviors and placing this on participants	“*My mom was a nurse and she’s very big on health, so I don’t think there was ever a month going by where she wasn’t on a new diet, so diet culture was very big in my house, with like Skinny Pop and special smoothies, that stuff was just constantly on me*” “*It came to the point where like the pantry was locked, so anytime there was food I would eat as much of whatever I could find, especially if it was something I wasn’t normally allowed to eat*”
	Positive Family Emphasis on Food (*n* = 19)		Participants described growing up in an environment where family mealtime was important, with an emphasis on togetherness and positive memories being associated with mealtime	“*We’ve always tried to do a family dinner every night to just kind of come together and reconnect, which is really nice. It’s definitely a social aspect, we make time for it even with my extended family*”
	Unhealthy Eating Environment (*n* = 16)		Participants felt they had no guidance on healthy eating or portion control or grew up in a family that relied on cheap, unhealthy foods due to budget constraints	*“I was given like no guidance on nutrition or portion sizes or things like that, so I just remember growing up eating huge, gigantic portions and just not understanding that that’s not good for me*”
	Negative Influence of Family Dinner (*n* = 9)		Participants associated family mealtime with negative memories, such as conflict or being pressured to finish their meal to the point of discomfort	“*It was always that I would try not to talk too much because my mom would attack anything you say or do if it’s not to her standards and then I would just want to finish it all up and then leave*”
Emergence of symptoms	Transition to College (*n* = 22)	Newfound Freedom	Participants felt that their symptoms began to emerge once they began college, partially due to the increased stress of the transition, and others feeling it was the widespread access to food. Participants expressed that the new ability to make their own food choices led them to overconsume unhealthy foods	“*It became me always seeking that moment of being able to eat and associating food with freedom and having some time to myself and being happy, so I began to pursue that more and it kind of spiraled out of control from there*”
	Pressures in School and Relationships (*n* = 14)		Participants noticed their symptoms emerge once they began to feel pressures to adhere to an ideal body type in middle school and high school. They often described comparing themselves to peers and often turned to foods for comfort at this time	“*I was in middle school, and I just kept thinking everyone else was better than me because I knew I was bigger, and food was there all the time, so I was just like ‘here’s my pal’*”
	Triggering Event (*n* = 19)		Participants could recall a specific event or situation that led to their emergence of symptoms. These included parental divorce, moving, the end of a sport, or the end of a relationship. Food provided stability for many participants during these uncertain times	“*My dad was always switching jobs so we would move state to state when it wasn’t really necessary, so I never had a stable environment, I wanted to have control over something so that’s a huge part of it*”
	Worsening Mental Health (*n* = 12)		Participants saw a direct correlation between when their depression or anxiety worsened, and their symptoms started to become a problem	“*I was diagnosed with depression, and I started having anxiety and all of that stuff and it just kind of became a crutch for me, honestly*”
	Unhealthy Relationship from the Start (*n* = 9)		Participants felt that they couldn’t remember when the symptoms started, but that they have always had problems with eating and with food	“*I wasn’t eating unhealthy foods in excess, but I would eat 7 bananas in one sitting…. we used to grow cherry tomatoes and I would eat 100 of them in one sitting. I know healthy food is good for you, but everything is supposed to be in moderation. I’ve never known moderation. I’ve known all the way or nothing so it’s a really bad thing”*

## Discussion

The aim of this study was to determine the impact of ACEs and other childhood influences on the development of food addiction in college-attending young adults. There was an overall 21.9% prevalence of food addiction in this sample, which is higher than some other studies examining food addiction in this population [5, 35]. Jahrami et al. found a 20.3% prevalence in females and 17.4% prevalence in males [45], and Sengor and Gezer found a 21.1% prevalence of high YFAS scores [34], which are more similar to the findings of the present study. There was a 24.2% prevalence of high ACEs in this study, which is again higher than other studies on the college-attending young adult population [46–49]. In previous investigations of psychosocial factors at the university used in this study, rates of mental health disorders and ACEs have been higher than other college populations [50–52]. This population resides in Appalachia, an area plagued by health disparities [53], which could provide an explanation for this trend.

Quantitative analysis showed that although ACEs were significantly correlated with food addiction. However, when controlling for other potential influences, ACEs did not significantly predict food addiction. The impact of ACEs on the development of food addiction in clinical populations has been found to be significant [10, 11, 18, 20]. ACEs have also been found to be a significant predictor of the development of eating disorders in the clinical population [26, 27] and emotional eating in the general population [12]. Comorbidities of depression or other mental health disorders are common in the food addiction population [8] and eating disorder population [26]. Depression caused a 233% increased risk of developing food addiction, adding evidence of mental health’s significant influence on eating behaviors. Depression, but not ACEs, being a significant predictor of the development of food addiction in this population was partially explained by qualitative results.

When participants were asked about their eating environment growing up, a variety of themes emerged. Many participants described a pressure to be healthy, which was characterized by an emphasis on diet culture, being conscious of weight, and maintaining a certain physique. There were many descriptions of restrictive eating environments, where parents or caregivers would limit the types of food allowed in the home, with some even putting locks on pantries so that participants could not access them. Some participants reflected that they believe this contributed to their current symptoms, where they now overindulged in certain foods due to being deprived of them in childhood. Participants who discussed diet culture and restrictive eating environments commonly brought up their mothers’ eating habits. Research has shown that maternal eating habits are a significant influence on child eating habits [54], especially on their daughters [55]. Additionally, Birch and Fisher found that when parents strictly control children’s food intake, this can cause the child to have strong preferences for high-fat, energy-dense foods [30]. A qualitative study on food addiction by Paterson et al. found similar participant descriptions of restrictive eating environments during childhood [56]. The effects of this environment were further elucidated in the present study when asked about their emergence of symptoms, where participants described entering college was when their symptoms appeared or worsened. This was credited to the newfound freedom of making their own food choices, especially by those who had been restricted growing up. Participants also cited the stress of the transition to college as being a contributing factor to their symptoms. The college environment has a widespread availability of hyper-palatable food [34] and the life-stage of emerging adulthood in this population has been shown to worsen mental health symptoms, especially since COVID-19 [51, 57]. This, combined with a restrictive eating environment in childhood, is one way that food addiction was shown to develop in this study.

Participants also described positive eating environments growing up, with an importance of family mealtime, and associated positive memories tied to food and eating. The possibility that individuals with food addiction turn to food due to its association with positive memories needs further exploration. Other eating environments described by participants were characterized by unhealthy and processed foods, and these findings were consistent with the findings from Paterson et al. [56]. Research has shown that early childhood eating habits can extend into adulthood [58]. This study shows that when combined with other factors, these unhealthy eating practices can develop into food addiction. Further, participants described a lack of guidance on healthy eating from parents or caretakers, which has been shown to be correlated with obesity [28]. Participants also described negative associations with family dinner, and the possibility of food becoming a source of anxiety for these participants needs further exploration. The worsening of mental health was another reason for the emergence of symptoms, which aligns with depression being a significant predictor in the quantitative results. Depression and anxiety are shown to be correlated with poorer diet quality [50, 59] as well as overweight and obesity [60]. These findings contribute to research on these topics, but also show that depression can cause the more severe outcome of food addiction, when combined with other influences.

There were several other themes developed from the emergence of symptoms. Participants described noticing their behaviors once they felt pressure from school and relationships, largely due to body image pressures. Studies have found that body image pressures and consistent comments about weight and shape a significant contributing factor to the development of eating disorders [61, 62]. Participants also discussed their symptoms emerging after triggering events, including parental divorce, moving homes, relationships ending, or other familial distress. The ACE questionnaire captures the experience of childhood trauma, containing items such as sexual or physical abuse, neglect, and household substance use disorders. These findings show that certain events, that are not captured by the ACE questionnaire, can contribute to the development of food addiction. Participants cited that food provided them stability or something to cope with during these stressful times, contributing to the understanding of why food addiction can develop after these life events. It is possible that the evidence for ACEs leading to substance use disorder is consistently seen because ACEs contain more severe instances of trauma. The lack of evidence on ACEs being a significant predictor of food addiction in this study points to the potential that less severe childhood events can cause food addiction, because food is readily available and needed to survive. In other words, it is a more accessible coping mechanism that may require less adverse life events to become dependent on. The other influences captured by qualitative analysis also point to this. However, this needs further exploration.

The impact of the COVID-19 pandemic on eating behaviors in a variety of populations is substantial [63]. Investigations on its impact on food addiction rates have consistently shown higher prevalence of food addiction [64, 65] and that food addiction symptoms and comorbidities were exacerbated during the pandemic [66]. Although the impact of COVID-19 was not measured within this study, future investigations can ask participants about its impact on their food addiction symptoms or eating behaviors.

## Strengths and limits

This study has several limitations. The use of cross-sectional data from one university cannot be generalized to the entire college-attending young adult population. Additionally, data was self-reported, which can result in respondent bias. Respondents to the survey were primarily female, and so were interview participants, although food addiction and eating disorders have been shown to occur in higher rates in females than males [45, 67]. This study also showed that being female was trending towards being a significant predictor of the development of food addiction. However, more investigation into food addiction in males is needed. This study also has several strengths. This is one of the few studies examining food addiction in the college-attending young adult population, which as in at-risk population. To our knowledge, this is the first mixed-methods study on food addiction in the non-clinical population, and one of the few that examines food addiction qualitatively in any population. Qualitative analysis in this study allowed for further exploration of contributing factors to the development of food addiction.

### What is already known on this subject?

ACEs are significant contributor to the development of food addiction in the clinical population. Some qualitative studies have investigated influences on the development of food addiction and found childhood exposure to diet culture to be a significant theme. College students have shown high rates of food addiction but investigations into causes in this population are minimal.

### What this study adds?

This study demonstrated that there are many factors contributing to the development of food addiction in the non-clinical population. Although ACEs contribute to the development of obesity and substance use disorder, other childhood influences, such as restrictive eating environments, are potential contributing factors to young adulthood food addiction. Further, the college environment, characterized by high stress and widespread availability of food, is when most participants’ symptoms developed. This adds evidence to the risk of development of food addiction in this population and highlights those resources and treatments for food addiction are needed on university campuses. Providing accessible counseling with therapists and dietitians is one potential way to treat this issue.

## Data Availability

Data can be made available upon request to the corresponding author.
